# Dunking into the Lipid Bilayer: How Direct Membrane Binding of Nucleoporins Can Contribute to Nuclear Pore Complex Structure and Assembly

**DOI:** 10.3390/cells10123601

**Published:** 2021-12-20

**Authors:** Mohamed Hamed, Wolfram Antonin

**Affiliations:** Institute of Biochemistry and Molecular Cell Biology, Medical School, RWTH Aachen University, 52074 Aachen, Germany; mhamed@ukaachen.de

**Keywords:** amphipathic helix, ALPS motif, membrane curvature, Nup155, Nup53, Y-complex, Nup153

## Abstract

Nuclear pore complexes (NPCs) mediate the selective and highly efficient transport between the cytoplasm and the nucleus. They are embedded in the two membrane structure of the nuclear envelope at sites where these two membranes are fused to pores. A few transmembrane proteins are an integral part of NPCs and thought to anchor these complexes in the nuclear envelope. In addition, a number of nucleoporins without membrane spanning domains interact with the pore membrane. Here we review our current knowledge of how these proteins interact with the membrane and how this interaction can contribute to NPC assembly, stability and function as well as shaping of the pore membrane.

## 1. Introduction

Nuclear pore complexes (NPCs) are the gatekeepers of the nucleus. They control the flux of molecules across the nuclear envelope, consisting of an outer and inner nuclear membrane, and accordingly establish the identity of the nucleus and cytoplasm as distinct compartments. While metabolites and ions freely diffuse through NPCs, the passage of larger molecules is constrained by their size and surface properties [1]. In contrast, selective nuclear import and export of macromolecules such as most proteins and RNA-protein complexes require transport signals. These are recognized by nuclear transport receptors (NTRs) that can alone or with their cargos rapidly cross NPCs by facilitated diffusion. In addition to their principal task in nuclear transport, NPCs have important functions in chromatin organization and gene regulation [2,3].

**Figure 1:**
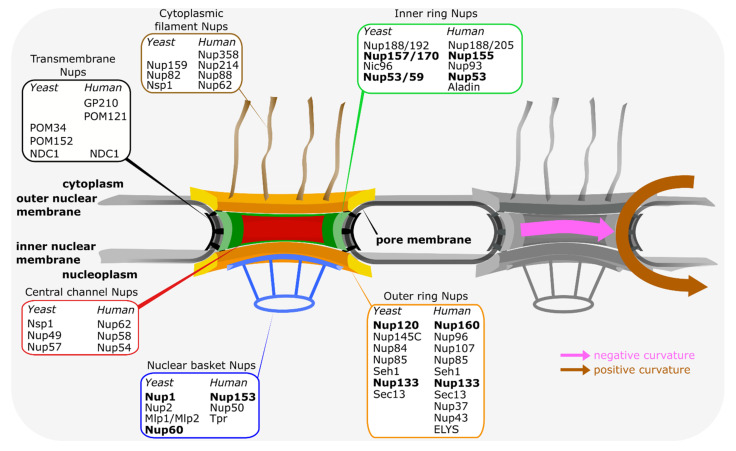
Main features of the vertebrate and budding yeast nuclear pore complexes. Schematic representation of the NPC, highlighting the ring and peripheral structures of the NPC. The inner ring (green) is sandwiched between the two outer rings, i.e., the nucleoplasmic and cytoplasmic rings (both in yellow/orange). From the outer rings, cytoplasmic filaments (brown) and the nuclear basket structure (blue) extend. The inner ring (green) is the main connecting point between the transmembrane nucleoporins (black) and the central channel nucleoporins (red), which are crucial for the transport and exclusion properties of the NPC. The distribution of nucleoporins, referring to the human and budding yeast nomenclature, within the different structural elements is indicated. Nucleoporins, which can directly interact with the pore membrane, are shown in bold. On the right, the positive and negative curvature of the pore membrane is indicated.

NPCs are embedded in the two membrane structure of the nuclear envelope at sites where these two membranes are fused [4,5]. With a mass of about 110 MDa, NPCs are the largest protein complexes in most vertebrate cells. In *Saccharomyces cerevisiae*, the approximate mass of 65 MDa is smaller but still considerable. In spite of their enormous size, NPCs are composed of about only 30 different proteins called nucleoporins (Figure 1), which are all present in multiple copies in these assemblies. These nucleoporins can be roughly categorized in three groups: first, phenylalanine-glycine (FG) repeat nucleoporins which are crucial for the transport and permeability properties of NPCs. They generally form an adhesive network in the center of the NPC and serve as docking points for NTRs. Second are structural nucleoporins which constitute the NPC framework. They are arranged in three prominent ring-shaped structures—the inner ring and two outer rings, the cytoplasmic and the nuclear ring. This three ring core encloses a central transport channel, formed mostly by the FG-repeat nucleoporins, and anchors extensions on the cytoplasmic and nuclear faces forming cytoplasmic filaments and nuclear basket, respectively. Structural nucleoporins are mostly composed of alpha-helical repeats or beta-propellers or a combination of both and show structural similarity to clathrin, COPI and COPII vesicle coating proteins [6,7,8,9]. Accordingly, the proto-coatomer hypothesis states that these nucleoporins act as NPC coats on the pore membrane and are, along with vesicle coating proteins, derived from a common ancestral membrane shaping unit [8,10,11]. This is further corroborated by proteins moonlighting between different coating functions such as Sec13 or Seh1, which are part of both the NPCs and the COPII or the SEA complex, a vacuolar membrane coat in yeast species. As a third group, transmembrane nucleoporins act as attachment points for scaffold nucleoporins to the pore membrane. Although the general structure of NPCs is evolutionarily conserved [5,12], at least in the organisms analyzed, individual nucleoporins often show little conservation between evolutionary distant organisms and only few nucleoporins are found in all species. For example, of the three vertebrate transmembrane nucleoporins, GP210, POM121 and NDC1, only NDC1 and POM121 are more widely conserved, but not found in all species [13,14,15]. Thus, additional anchoring points for nucleoporins to the pore membrane must exist. Although it cannot be excluded that additional transmembrane nucleoporins have escaped our attention, direct membrane interactions of proteins without membrane spanning regions thus must contribute to the linking of the NPC to the pore membrane. Indeed, a number of nucleoporins show direct membrane binding and we will review here how they contribute to NPC structure and its assembly.

## 2. Modes of NPC Assembly

NPCs assemble and integrate into the membranes of the nuclear envelope. For this, the shaping of membranes is crucial and direct membrane interactions of nucleoporins critically contribute, as discussed below. Depending on whether cells break down their nuclear envelope during mitosis or not, different modes of NPC assembly exist: animal cells typically proceed via an open mitosis and disassemble the nuclear envelope, at least partially, with entry into mitosis and reassemble the nuclear envelope after successful chromatin segregation. In this mode, NPCs disassemble with the nuclear envelope at the beginning of mitosis and reassemble with the nuclear envelope during late anaphase and telophase, referred to as postmitotic or mitotic NPC assembly [16]. In addition, NPCs form de novo throughout interphase, which is hence referred to as interphase NPC assembly. Mitotic NPC assembly is rather fast with high numbers of NPCs assembling fairly synchronously [17]. In contrast, interphase NPC assembly is, at least in vertebrate cells, rather slow and sporadic [18]. Whereas interphase NPC assembly integrates NPCs into the intact double membrane layer of the nuclear envelope and therefore requires fusion of the inner and outer nuclear membrane, it is a matter of debate whether mitotic NPC assembly follows the same route, i.e., insertion into a double membrane, or whether it bypasses the need of inner-outer nuclear membrane fusion by assembling into already existing membrane holes or engulfment on the chromatin surface, assembling NPCs via the reforming nuclear envelope [19,20].

In cells, which keep the nuclear envelope largely intact during mitosis (closed mitosis), NPCs inevitably integrate into the intact nuclear envelope. This follows the route outlined for interphase NPC assembly.

## 3. Lipid Composition of the Nuclear Envelope

The membrane of the nuclear envelope forms a membrane continuum with the endoplasmic reticulum (ER) and can therefore be considered as a subdomain of the latter. The inner nuclear membrane possesses a protein composition distinct from the outer nuclear membrane and the other parts of the ER, established and maintained by active transport of some inner nuclear membrane proteins and/or binding to chromatin and the lamina [21]. In contrast, the lipid composition of the outer and inner nuclear membrane largely resembles that of the other ER domains. The mammalian nuclear membranes contain approximately 60% phosphatidylcholines (mole fraction of the lipids), 50% more than the plasma membrane but less lysophosphatidyl-choline, sphingomyelin, and cholesterol [22,23,24]. Similarly, the *S. cerevisiae* ER-membranes are rich in phosphatidylcholines [25]. Phosphatidylcholines in animal and yeast species possess frequently unsaturated and thus kinked fatty acid side chains while the acid chains of sphingomyelin and lysophosphatidyl-choline are commonly saturated. This results in a higher lipid packing density at the plasma membrane, further enhanced by the higher cholesterol content, which can act as a molecular glue between the phospholipids [26]. The ER membrane including the inner and outer nuclear membranes is softer and more fluid than the plasma membrane. ER membrane lipids are more loosely packed so that their hydrophobic tails are transiently exposed to the solvent creating spaces between the lipid head groups. These lipid packing defects favor membrane interactions of peripheral proteins via hydrophobic insertions such as amphipathic helices, a mode prevalent in the membranes of the early secretory pathway [26] including the pore membrane (see below). In contrast, plasma membrane binding of proteins more frequently relies on charge–charge interactions enabled by the negative surface charge of the cytosolic face of the plasma membrane caused by the asymmetric distribution of phosphatidylserines.

## 4. Nucleoporin Interactions with the Nuclear Pore Membrane

### 4.1. Y-Complexes Interact with the Pore Membrane Via ALPS Motifs

One of the main architectural elements of the NPC is the Y-complex, named after its Y shape [27], also referred to as coat nucleoporin complex, Nup84-complex in *S. cerevisiae* and Nup107-Nup160 complex in vertebrates. In *S. cerevisiae*, it is a heptameric 575 kDa complex composed of Nup84p, Nup85p, Nup120p, Nup133p, Nup145Cp, Sec13p and Seh1p. In metazoan, three additional proteins, Nup37, Nup43 and ELYS/MEL28, can be part of the complex, where Nup107 corresponds to Nup84, Nup96 to Nup145C, and Nup160 to Nup120 [14,28,29]. Multiple copies of the Y-complexes arrange into annular assemblies forming the largest part of the cytoplasmic and nucleoplasmic ring structures of the NPC. Although components of the Y-complexes can be found in all eukaryotes [13,14], the precise arrangement varies between species. In *S. cerevisiae*, the cytoplasmic and nucleoplasmic rings are each composed of eight Y-complexes, which arrange in a head to tail fashion to form these rings [30,31]. In human cells, Y-complexes also dimerize with themselves resulting in a double-ring arrangement within each of the cytoplasmic and nucleoplasmic rings, totaling 32 Y-complexes per NPC [32]. In other species, hybrids of both arrangements are observed: in the algae *Chlamydomonas reinhardtii* the nuclear ring consists of 16 Y-complexes but the cytoplasmic ring only of eight [33]. In the fission yeast *Schizosaccharomyces pombe*, the nuclear ring also consists of 16 Y-complexes and eight Y-complexes localize to the cytoplasmic site of the NPC which are, however, not connected to a ring-like arrangement [34,35].

The nucleoporins forming the Y-complex are, as most structural nucleoporins, mainly composed or alpha-helical repeats or beta-propellers, or a combination of both. As other structural nucleoporins, they show structural similarity to vesicle coating proteins [7,8,9] and share a common evolutionary ancestry with these [10]. COPI, COPII and clathrin coats interact with their target membrane via transmembrane proteins [36] and, for the vertebrate Y-complex biochemical data, show an interaction to POM121 [37], a single spanning transmembrane nucleoporin facing with its largest parts towards the NPC [38]. Yet, the current cryo-EM reconstructions of the NPCs fail to localize POM121 [39,40,41].

**Figure 2:**
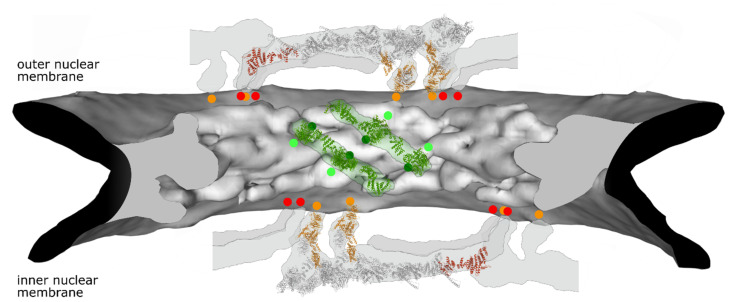
Selected membrane contact sites within the NPC structure model. Electron density of the NPC inner ring is shown in grey (generated from PDB:7PER). The position of four Nup155 molecules in green as well as their membrane contact sites (light green dots) is shown. Predicted Nup53 membrane contact sites (dark green dots, based on [39]) are similarly indicated. For the outer rings (generated from PDB: 7PEQ) the position of two complete Y-complexes with parts of their neighboring units are shown. Nup160 (orange) and Nup133 (red) with the respective membrane contact points are indicated. Please note that the position of the second Nup133 in one of the Y-complexes as well as the position of Nup53 in the inner ring cannot be unambiguously assigned in this structural model. Similarly, the current NPC structure models do not show the position of Nup153.

**Figure 3:**
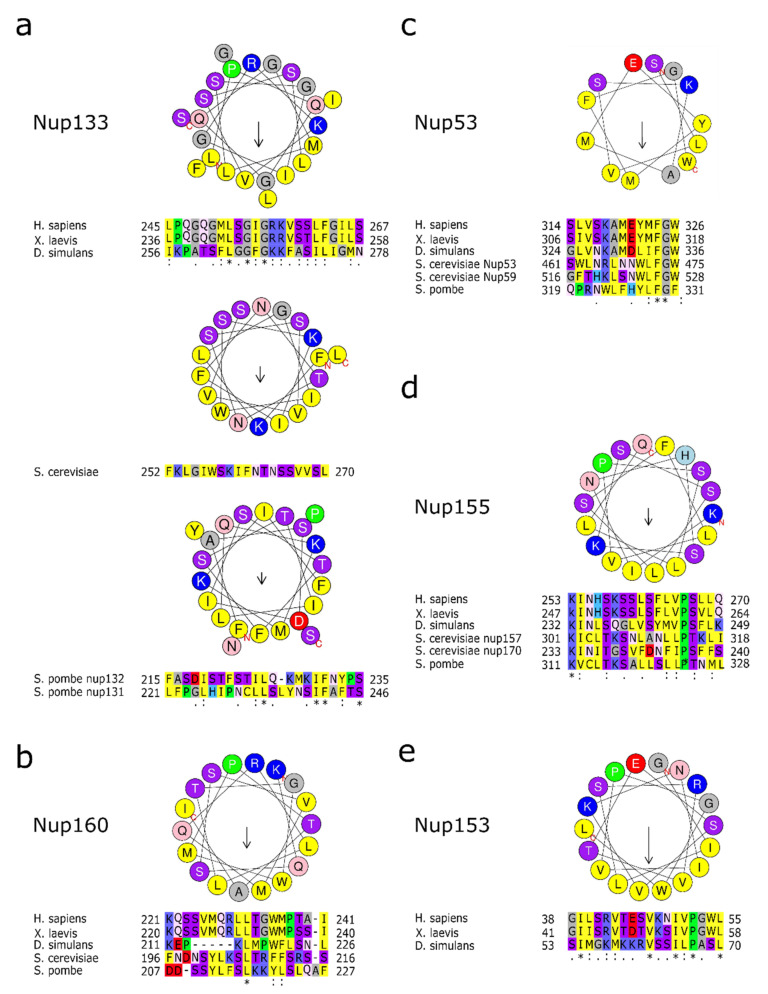
Membrane binding amphipathic helixes in nucleoporins. (**a**) Amino acid sequence of human Nup133 amphipathic helix as well as the *S. cerevisiae* and *S. pombe* orthologues in a helical wheel representation. Below are the alignments of the corresponding sequences in metazoan or the two *S. pombe* orthologues as well as the *S. cerevisiae* sequence. (**b**) Helical wheel representation of human Nup160 amphipathic helix. Below are the alignments of the corresponding sequences in metazoan or yeast species. (**c**) Helical wheel representation of human Nup53 amphipathic helix. Below are the alignments of the corresponding sequences in metazoan, the *S. cerevisiae* Nup53 and Nup59 sequences and Nup53 from *S. pombe*. (**d**) Helical wheel representation of human Nup155 amphipathic helix with the alignments of the corresponding sequences in metazoan, the corresponding Nup157 and Nup170 sequences from *S. cerevisiae* and Nup155 from *S. pombe*. (**e**) Human Nup153 amphipathic helix in a helical wheel representation. Below are the alignments of the corresponding sequences in metazoan. Please note that the sequence of the membrane interaction motif is not conserved in the yeast orthologues, which nevertheless can directly interact with membranes. Helical wheel representation was generated with HeliQuest [43], where the length of the arrow within the helix is proportional to the mean hydrophobic moment. Asterix (*) indicates positions with fully conserved amino acid residues, colon (:) indicates conservation between amino acid residues of similar properties, period (.) indicates conservation between amino acid residues of weakly similar properties.

In addition to interactions via transmembrane nucleoporins, the Y-complexes directly contact the pore membrane at several points (Figure 2). They do so via membrane binding motifs known as ALPS motifs, which are mainly located at the tips of Nup133 and Nup120/Nup160 facing the nuclear membranes (Figure 3). For Nup133, a membrane binding sequence, an ALPS motif, was first identified in the human protein [42]. ALPS, for ArfGAP1 lipid packing sensor, motifs are 20–40 amino acid stretches in proteins targeted to curved regions of membranes. They are unfolded in solution but adopt an amphipathic helix conformation in the presence of curved membranes, so that the hydrophobic surface of the helix interacts with the membrane. These helices lack a clear sequence homology between different proteins but share some common features [42,43]: they possess only few charges on their polar face, which is mainly composed of glycine, serine and threonine residues. Proteins with ALPS motifs often localize at membranes with low content of negatively charged lipids, like the ER network and the cis-Golgi apparatus, and thus rely mainly on hydrophobic interactions to partition into the bilayer. In the case of Nup133, the ALPS motif loops out from the N-terminal beta propeller structure, and appears unstructured in the crystal structure of the human Nup133 N-terminal domain [44]. This domain binds pure lipids vesicles, liposomes, in membrane floatation assays, indicating that the ALPS motif can indeed interact with membranes [42].

While the Nup133 ALPS sequence is highly conserved in metazoan, it has been debated whether this motif is also present in yeasts [44]. The structure of *Vanderwaltozyma polyspora* Nup133 N-terminal domain suggested a loop in the same region as in human Nup133 and structural modeling proposed one ALPS motif in *S. cerevisiae* Nup133 [45]. Indeed, the recently solved crystal structure shows an unresolved loop region in the same position [46]. Accordingly, the protein fragment binds liposomes in membrane floatation assays.

In human U2OS cells, a fusion protein where the Nup133 ALPS motif was introduced into EGFP localizes to tubular ER structures and small liposomes, both indicating a preference for highly curved membranes [47,48]. Liposome binding and ER-membrane localization is lost when a negative charge is introduced into the hydrophobic surface of the ALPS motif. Interestingly, the electrostatic or hydrophobic content of the Nup133 ALPS modifies the avidity for cellular membrane but not the organelle targeting properties [47]. In the Nup133 structures, the ALPS motif is constrained between two beta sheets of the N-terminal beta-propeller domain. In the absence of these restrains, the ER tubule localization of the Nup133-ALPS is lost [47]. Interestingly, when anti-GFP antibody coated beads were injected into GFP-Nup133 expressing HeLa cells, which generate GFP-Nup133 coated beads in these cells, nuclear envelope-like membrane structures formed on these beads [49]. Whether this is due to Nup133 membrane binding capabilities and especially the ALPS motif or mediated by other factors, e.g., other nucleoporins of the Y-complex binding to Nup133, remains open.

Functional studies in U2OS cells suggest that the human Nup133 ALPS motif is important for interphase but not mitotic NPC assembly and its membrane binding feature seems crucial for its function in interphase assembly [48]. However, it remains unclear whether this function is required in all cell types: Nup133 is essential for mouse development beyond gastrulation [50]. However, in mouse embryonic stem cells Nup133 is not necessary for cell growth at the pluripotent stage [50]. In these cells, the NPC scaffold can assemble in the absence of Nup133 and Nup133 in mouse embryonic stem cells is not required for interphase NPC assembly [51]. In *S. cerevisiae* [52], but also *S. pombe* [53], *Aspergillus. nidulans* [54], *Lotus japonicus* [55] and *Caenorhabditis elegans* [56] Nup133 is dispensable for cell viability.

Although *S. cerevisiae* Nup133 can bind membranes in liposome floatation assays [46], it remains unclear how the deletion of the ALPS motif would impact Nup133 targeting and NPC structure and function in this organism. Phenotypic heat maps show that Nup133 mutations lead to NPC clustering, which has been interpreted as a membrane interaction phenotype [57]. Nup133 versions lacking the N-terminal domain cannot complement the NPC clustering phenotype [52] which would be consistent with the idea that membrane interaction of Nup133 is required for proper NPC spacing.

It is possible that other parts of the Y-complex compensate for the loss of Nup133 for membrane binding. Indeed, structural modeling suggests that *S. cerevisiae* Nup120 contains two ALPS motifs [42,45]. Similar to Nup133, Nup120 mutations lead to a NPC clustering phenotype [57]. The metazoan orthologue Nup160, has been suggested to contact the pore membrane similarly via its beta-propeller [41,58]. Furthermore, in vertebrates, Nup153, a direct interaction partner of the Y-complex, binds in interphase NPC assembly to the inner nuclear membrane and recruits the Y-complex (see below). It is therefore possible that loss of Nup133 function might be compensated for by this interaction. In addition, interaction of POM121 to the remaining Y-complex might bypass the need for Nup133.

### 4.2. Nup53 and Nup59

Nup53, in metazoan also referred to as Nup35, is part of the inner ring complex (IRC), also known as Nup93 complex [59], which serves as an important linker between the transmembrane nucleoporins and the central channel. In this respect, vertebrate Nup53 interacts with NDC1, a nucleoporin with six transmembrane regions and a C-terminal domain facing towards the NPC [60,61], and Nup93 [62,63], which in turn binds the Nup62-complex (composed of Nup62, Nup58, Nup54/45) of the central channel [64,65]. It also interacts with Nup155 [66,67], which can in turn bind POM121 and NDC1 and thus serves as a second membrane anchorage point of the IRC [37,68]. In *S. cerevisiae*, the two orthologues, Nup53p and Nup59p, similarly interact with Ndc1p and via Nic96p, the Nup93 orthologue, with the Nsp1-complex corresponding to the Nup62-complex [69,70,71,72,73,74,75].

*S. cerevisiae* Nup53p has a predicted C-terminal amphipathic helix (Figure 3), which has been suggested to serve as membrane interaction motif [76,77]. Indeed, overexpression of the Nup53p results in massive nuclear envelope proliferation, an effect which is dependent on this motif [76]. In biochemical assays, both yeast Nup53p and Nup59p float with liposomes and tubulate membranes, which was similarly seen for vertebrate Nup53 [78]. In vertebrate Nup53, two membrane interaction motifs were identified: within the N-terminus, a basic motif which presumably relies on charge-charge interactions with the membrane and the C-terminal amphipathic helix, similar to the ones described in yeast Nup53p and Nup59p [78]. In vitro studies show that at the end of mitosis, either one of the two membrane interaction motifs is sufficient for NPC assembly whereas for interphase NPC assembly, the C-terminal amphipathic helix is strictly required. This could indicate that for both assembly modes Nup53 needs to bind directly to the nuclear envelope membranes but that for interphase NPC assembly specific features of the amphipathic helix, speculatively its membrane bending activity, are required. For efficient membrane interaction and NPC assembly, dimerization of vertebrate Nup53 is required, probably by an avidity effect. In *C. elegans*, the Nup53 dimerization domain is similarly crucial for its function in NPC assembly [79]. Since the dimerization interface found in the mouse protein is evolutionarily conserved [80] dimerization might be a characteristic of most Nup53 orthologues but is not necessarily found in all organisms as *Chaetonium thermophilum* Nup53 is monomeric in solution [71].

Despite its capability to directly bind membranes, vertebrate Nup53 interacts with the transmembrane nucleoporins POM121 and NDC1 [37,60,66], and the latter interaction is also seen in *S. cerevisiae* [70]. Interestingly, in vertebrates, CDK1 mediated phosphorylation of Nup53 breaks up this interaction, an event needed for efficient NPC disassembly at the beginning of mitosis [81]. The N-terminal membrane binding site of Nup53 can also be phosphorylated by CDK1, which, at least in vitro, prevents membrane interaction of this motif [78]. Thus, it is likely that breaking membrane interaction of Nup53, direct or indirect via NDC1, drives NPC disassembly. In turn, integrity of these interactions is required for the assembled state of the NPC.

The C-terminal membrane binding site of Nup53 is in close proximity to the NDC1 interaction site [60,66], while NDC1 requires its N-terminal part, composed of the six transmembrane regions for this interaction [66]. In vitro studies suggest that the NDC1-Nup53 interaction serves as more than an additional membrane anchorage point of the IRC [66]. While NDC1 is required for NPC assembly in the presence of full-length Nup53, the N-terminal domain of NDC1 and thus its ability to directly interact with Nup53 becomes dispensable if Nup53 lacks its C-terminal membrane interaction motif. As this motif possess membrane curving activity it might be speculated that it is this feature which is controlled by the NDC1 interaction. In the NPC assembly process the membrane faces changes in curvature where an exceeding curvature induction might need to be balanced or prevented [66].

### 4.3. Nup155 and Its Orthologues Nup157 and Nup170

Nup133, Nup160 and Nup155 share a similar arrangement composed of a beta-propeller followed by an alpha-helical region, suggesting they are derived from a common ancestral membrane modeling unit [82]. Similar to Nup133 und Nup160, a loop region of the Nup155 beta-propeller region dips into the lipid bilayer [41]. Cryo-electron tomography of the human NPC combined with structural modeling places this domain at the pore membrane (Figure 2). Liposome floatation assays suggest that the loop region indeed functions as membrane binding module. Nup155 mutants defective in membrane binding cannot replace the endogenous protein in NPC assembly suggesting a crucial role of this motif for NPC assembly and/or structure [83].

Modeling of the *S. cerevisiae* Nup157p/Nup170p structures into the human NPC structure model suggested that the membrane binding motifs are conserved [84] (Figure 3). Indeed, the current model of the *S. cerevisiae* NPC places the beta-propellers of Nup157/Nup170 at the pore membrane [30]. The direct interaction of Nup53 with Nup155 or Nup53p/Nup59p with Nup157p/Nup170p in *S. cerevisiae*, might further enhance membrane interaction. As the Nup155 binding site within Nup53 is close to the C-terminal membrane binding motif it has been argued that this arrangement, also found in *S. cerevisiae* and *C. thermophilum*, clusters the membrane binding motifs [84]. Dimerization of Nup53 might, where present, further enhance this effect. The multiple interaction sites with the pore membrane might also cause a degree of redundancy and robustness: in *S. cerevisiae*, a double deletion of Nup53 and Nup59 is viable. The co-depletion with Nup157 causes severe growth defects, and only the co-depletion with Nup170 is lethal [69]. This redundancy might also explain why no orthologues for Nup53/59 have been detected in *A. nidulans*, but where Nup170 is essential [54].

### 4.4. Nup153 and Relatives, Membrane Interacting Nucleoporins of the Nuclear Basket

Nup153 is a central part of the nuclear basket structure of metazoan NPCs with important functions in mRNA export [85,86,87]. The protein interacts with the two other basket components, Nup50 and TPR [88,89] and the Y-complex and thus likely links the basket to the nuclear ring [90]. Nup153 possess an amphipathic helix in the N-terminal domain [91]. This domain binds membranes in liposome floatation assays and the inner nuclear membrane if expressed on cells, and both features depend on the amphipathic helix [86,91]. Interestingly, the NTR transportin also binds Nup153 N-terminal domain and this interaction prevents liposome binding. Similar as described for Nup133, injection of GFP antibody coated beads into GFP-Nup153 expressing HeLa cells generates nuclear envelope-like membrane structures on theses beads [49]. Here also, it remains open whether the membrane binding motif of Nup153 is required for this.

Functionally, Nup153 is required for interphase but not mitotic NPC assembly and its membrane binding ability is crucial for its function in interphase assembly [91]. In this process, it binds to the inner nuclear membrane and recruits the Y-complex to the newly assembling NPC. The membrane bending activity of amphipathic helix is seemingly not crucially required for this process as its function can be bypassed by directly recruiting the Y-complex to the inner nuclear membrane via a transmembrane protein, at least in vitro. Transportin is suggested to bind newly synthesized Nup153 in the cytosol preventing membrane interaction and guiding the protein into the nucleus. Here, release from transportin allows Nup153 to interact with the inner nuclear membrane to fulfill its function in NPC assembly.

The *S. cerevisiae* Nup153 orthologues Nup1 and Nup60 both possess an amphipathic helix in the N-terminus [92]. These helices, fused to mCherry, bind membranes in liposome pelleting assays and bend them, but their amphipathic helixes are not classical ALPS motifs as they contain charged amino acids and bind liposomes of all sizes. Nup60 membrane interaction was previously reported using liposome binding to Nup60p-covered beads [77]. Overexpression of the N-terminal domains of either Nup1 and Nup60 causes nuclear envelope expansion and deformation [92], reminiscent of Nup53 overexpression [76]. Surprisingly, combined deletion of the amphipathic helixes in both proteins did not severely affect growth of yeast cells suggesting that Y-complex recruitment to the nuclear membrane and assembling NPCs might follow alternative or redundant routes [93]. However, the amphipathic helixes of Nup1 and Nup60 become vital when the otherwise non-essential transmembrane nucleoporin Pom34 is deleted suggesting that the Nup1 and Nup60 amphipathic helixes together with other membrane shaping factors are needed for NPC assembly and maintenance.

### 4.5. Nup50 and Nup2, Nuclear Basket Nucleoporins as Phosphoinositide Binders

Nup50 and its *S. cerevisiae* orthologue Nup2 are also part of the nuclear basket structure of NPCs. Nup50/Nup2 interact with NTRs and the small GTPase Ran supporting the nuclear transport [94,95]. *S. cerevisiae* Nup2 possess a predicted PH2 (Pleckstrin homology 2) domain overlapping with its Ran binding domain [96]. Indeed, a recombinant fragment of Nup2 comprising this region binds especially phosphatidylinositol-4,5-bisphosphate (PI(4,5)P2) or phosphatidylinositol-3,4,5-trisphosphate (PI(3,4,5)P3) liposomes. Consistent with this, the vertebrate orthologue Nup50 has been identified as a PI(4,5)P2-interacting protein [97]. The functional implication of these interactions, both in yeast and vertebrates, is unclear. Given that PI(4,5)P2 and PI(3,4,5)P3 are highly abundant in the nucleoplasm [98], at least in vertebrate cells, it remains open whether these interactions direct Nup50 to the nuclear envelope. Indeed, a large fraction of Nup50 is found in the nucleoplasm [99,100,101] but also here it remains open whether Nup50 interacts with the nuclear phospho-inositides and whether this affects its function(s). In vertebrates, Nup50 truncations lacking the predicted phosphoinositide binding site still localize to NPCs and fulfil their crucial function in mitotic NPC assembly [101].

## 5. Membrane Interactions for Curvature Stabilization or Induction?

Since NPCs are located at the sites of fusion between the inner and outer nuclear membranes, it has been argued that they need to stabilize the strong curvature of the pore membrane [5,102]. This function is analogous to those of vesicle coats, but the pore membrane topology with its saddle-like appearance is different to vesicle membranes owing to the presence of both negative and positive membrane curvature [103]: it possess a concave shape (negative curvature) in the plane of the pore and a convex shape (positive curvature) along the membrane connection form the outer to the inner nuclear membrane (Figure 1). However, calculations indicate that positive and negative curvature can at least partially balance each other [104]. Indeed, recent molecular dynamics simulations suggest that a pore in a double membrane structure could be stable in the absence of proteins, albeit with a much smaller diameter than the NPC [39]. The NPC scaffold may thus not be needed to stabilize the membrane hole per se, but rather functions to widen the diameter of the pore. As these calculations are based on pure lipid bilayers, it will be interesting to learn to which extent they reflect the natural situation of biological membranes in the context of a nuclear envelope.

If membrane bending is nevertheless needed membrane binding nucleoporins could contribute in two ways to curvature generation. Amphipathic helixes as found in nucleoporins can band membranes by inserting into one leaflet of the lipid bilayer and functioning as a wedge [102,105]. Because of the surface expansion of this leaflet in relation to the other leaflet, the membrane adopts a positive curvature. Indeed, experimental work [106] and calculations [107,108] indicate that amphipathic helix insertion into lipid membrane is an efficient way for curvature generation. This has also been demonstrated for Nup53/Nup53p/Nup59p and Nup153/Nup60p/Nup2p [78,91,92]. Furthermore, amphipathic helixes might act, in addition to interactions with transmembrane proteins, as membrane anchoring points for scaffolding nucleoporins functioning as membrane deforming coats. The oligomerization of Y-complexes and IRC components to annular assemblies and their structural similarity to membrane coating proteins underscores this possibility. Certainly, both mechanisms are non-exclusive and could synergize for pore membrane bending.

Only a subfraction of nucleoporins in the outer and inner rings of the human and *S. cerevisiae* NPC have direct contact with the nuclear envelope [30,41,109,110] but because they are present in multiple copies this becomes a considerable number. Transmembrane nucleoporins are located in the nuclear envelope and act as further contact points, but their structures, except for the luminal parts of GP210 and POM152p, and precise localization within the NPC have not yet been elucidated. Within the assembled structures, the detectable contacts between the Y-complexes and the nuclear envelope are mediated by the ALPS motif-containing Nup160 and Nup133 beta-propellers. As 32 Y-complexes are present in the vertebrate NPC, this adds up to 64 possible membrane contact sites. In *S. cerevisiae*, because of the reduced number of Y-complexes, 32 possible membrane contacts are still contributed by Y-complexes. Within the inner ring complex, 32 Nup155 or Nup157p/Nup170p form the scaffold layer closest to the pore membrane, and they also contact the nuclear envelope through their ALPS motif-containing beta-propellers. The inner and outer rings are connected in humans and *C. reinhardtii* by an additional group of 16 Nup155 bridging molecules, which also contact the membrane with their ALPS motif-containing beta-propellers [33,58]. Nup53 in humans and Nup53p/Nup59p in *S. cerevisiae* are present in 32 copies.

These many potential membrane contact sites are not evenly distributed on the pore membrane. As discussed, Nup155 and Nup53 can interact so that the membrane interaction motifs of the two nucleoporins might act in concert, further enhanced by the Nup53 dimerization, where present. Similarly, membrane interactions of Nup133 and Nup160, both part of the Y-complexes, have been suggested to be clustered [5]. As Nup153 interacts with Y-complexes of the nuclear outer ring, this might further contribute, although Nup153 is not assigned in the current NPC structures [39,41]. Thus, multiple membrane-binding motifs can be used simultaneously at each membrane contact site. This arrangement is reminiscent of other membrane-bending coats, where coat proteins similarly form highly clustered membrane contacts [5]. This clustering might also explain the robustness of the NPC structure with regard of loss of certain nucleoporins, e.g., Nup133 [51,52,53,54] and Nup53/59 in budding and fission yeast [69,111]. Recent cryo-EM structures of NPCs lacking the cytoplasmic ring or both the cytoplasmic and nuclear rings, induced by degron-mediated degradation of the Y-complex component Nup96, show the inner ring embedded in the nuclear envelope with a smaller distance between outer and inner nuclear membrane and thus a higher positive curvature along their connection [40]. Thus, the Nup160 and Nup133 mediated membrane interactions are in this situation not required to maintain the membrane pore structure. Interestingly, this study shows in the untreated condition an asymmetric membrane shape along the outer and inner nuclear membrane connection with a higher curvature at the nuclear site. It remains open whether this is due to asymmetric distribution of membrane bending modules (e.g., Nup153 acting on the nuclear site), asymmetric interactions between different outer and inner ring nucleoporins with transmembrane nucleoporins, asymmetric localization of the latter, or other causes.

Membrane contact sites are not only important for stabilizing the intact NPC. They contribute also to NPC assembly. As discussed, in vertebrates membrane binding of Nup153 and the C-terminal membrane binding motif of Nup53 specifically contribute to interphase NPC assembly. Mitotic NPC assembly is possible without Nup153 and thus its amphipathic helix. Similarly, Nup53 truncations lacking the C-terminal membrane binding site still support mitotic NPC assembly. In these cases it can be concluded that these sites are not essential for stabilizing the NPC structure in the nuclear envelope but are specifically required for assembly of NPCs into an intact nuclear envelope. For other crucial membrane binding motifs, e.g., of Nup155, it is still open whether they are necessary for NPC assembly and/or stability. The amphipathic helixes involved could act by generating membrane curvature required for NPC formation or sensing an already curved membrane to direct nucleoporins to this sites for NPC assembly. However, because curvature recognition and generation by amphipathic helices rely on the same biophysical principles, the distinction between membrane shaping and curvature sensing is fluid [112].

Recent work in human tissue culture cells suggests that a subtraction of the Y-complexes and the inner ring complexes remain membrane-associated throughout the open mitosis, probably even as ring-shaped assemblies [113]. Although direct evidence is lacking, such arrangement could function as membrane located template for NPC reformation at the end of mitosis. If relevant, direct membrane interactions of Nup133 and Nup160 within the Y-complex as well as Nup155 and Nup53 in the inner ring complex might, together with interaction to transmembrane nucleoporins, be central for this.

In addition to nucleoporins, other membrane and membrane associated proteins affecting membrane curvature contribute to NPC assembly even if they are not part of the final NPC structure. Wedge-shaped membrane proteins important for the highly curved membrane structure of the tubular ER such as reticulons and REEP4 are involved in interphase NPC assembly [114,115]. In *S. cerevisiae*, the nuclear envelope transmembrane protein APQ12 is suggested to support NPC biogenesis by promoting phosphatidic acid accumulation with the help of an amphipathic helix, which would induce membrane curvature for the assembly process [116]. The *S. cerevisiae* NPC transmembrane protein POM33 possess two amphipathic helixes in the C-terminus which contribute to NPC targeting [117], probably by sensing the curved pore membrane. A supportive function for NPC biogenesis, akin to APQ12, cannot be excluded. The amphipathic helix is also predicted for a *S. cerevisiae* orthologue of Pom33, Pem33, and the *S. pombe* and metazoan homologues Tts1 and TMEM33.

## 6. Assays for Membrane Binding

Thus, a number of nucleoporins, which can directly and dynamically interact with membranes, have been identified (see Table 1). Membrane binding experiments are supported by the current NPC structures, which place the respective parts of the nucleoporins next to the pore membrane [39,41,84]. Additionally, functional studies validate the importance of the respective membrane interaction motif for NPC assembly [78,83,91,92]. Microscopic assays show nucleoporin-lipid-bilayer interactions using small (30 nm to 400 nm) liposome recruitment to bead bound proteins [48,77] or protein recruitment to large vesicles (giant unilamellar vesicles, up to 50 µm) [91]. Biochemical assays such as liposome floatation [42,46,78,91] or pelleting assays [92] allow an estimation of the liposome bound protein fraction. While in flotation assays the membrane binding protein is found together with the liposomes in the top fraction of a density gradient, pelleting assays rely on co-sedimentation of membrane binding proteins with liposomes, which can also occur due to protein aggregation and needs to be distinguished from the latter. Unfortunately, the different assays used complicate the comparison of membrane binding capabilities of the nucleoporins studied. Furthermore, liposomes employed differ in their lipid composition, e.g., containing complex lipid mixtures such as *E. coli* [78] or *S. cerevisiae* [46] polar lipids, lipid extracts from mammalian brain [78,92], well defined lipid mixtures with *S. cerevisiae* ER-like [77], vertebrate nuclear envelope-like [41,78,91] or Golgi-like [42] lipid compositions or an artificial membrane composition employing phosphatidylcholine as single lipid species [41,48,91]. These different assay conditions may explain in part the discrepancies observed: Nup133 has been reported to bind preferentially to small rather than larger liposomes [42,48,92], which is consistent with an ALPS motif which should preferentially interact with the highly curved membranes of smaller liposomes. However, other studies did not observe such a pronounced size dependence [91].

## 7. Conclusions and Outlook

In summary, the NPC protein network contacts the pore membrane at numerous sites, often via amphipathic helixes that insert into one lipid leaflet of the membrane. This could directly contribute to generation of pore membrane curvature or function to anchor the respective nucleoporins to the membrane. As both potential tasks go hand in hand, they are experimentally difficult to distinguish. It also remains open as to how many membrane interaction sites are minimally needed to generate and stabilize the pore membrane structure. The recent cryo-EM structure of NPCs lacking one or both outer rings and thus presumably the multiple Y-complex-membrane interaction sites indicates that a pore structure is stable with drastically reduced numbers of nucleoporin-membrane interactions [40]. Predictably, further studies of NPCs lacking specific nucleoporins and NPC substructures will reveal which membrane interactions are indeed crucial for NPC stability and/or assembly.

## Figures and Tables

**Table 1:** Nucleoporins acting as peripheral membrane proteins.

Nucleoporin	Main Functions	Nucleoporin Interactions
Nup133	part of the Y-complex [27] mRNA export [52,90] interphase NPC assembly [48] neural differentiation [50] proper NPC spacing in budding yeast [52,57]	binds within the Y-complex Nup84 and is also important for inter-Y-complex interactions [4,5]. The Y-complex acts as a central NPC building block and shows within NPCs manifold interactions, e.g., with Nup98, Nup153, POM121 [37,68,90]
Nup160 (Nup120 in budding yeast)	part of the Y-complex [27] mRNA export [90,118] proper NPC spacing in budding yeast [57,118]	binds within the Y-complex Nup85 and Nup96 (Nup145C in budding yeast) [4,5]
Nup53 (Nup53 and Nup59 in budding yeast)	part of the inner ring complex [4,5] mitotic and interphase NPC assembly [62,78]	NDC1, Nup155, Nup93 (NDC1, Nup157/Nup170 and Nic96 in budding yeast) [60,62,63,66,67,69,70,72,75,110]
Nup155 (Nup157 and Nup170 in budding yeast)	part of the inner ring complex [4,5] NPC assembly [119,120] mRNA export [121] gene silencing [122]	NDC1, POM121, Nup53, Nup93 (NDC1, Nup53/Nup59 and Nic96 in budding yeast) [37,66,67,69,70,72,110]
Nup153 (corresponds to Nup1 and Nup60 in budding yeast)	part of the nuclear basket [4] interphase NPC assembly [91] mRNA export [85,86] nuclear import [123] proper NPC spacing [124]	Y-complex [90] Nup50, TPR [88]
